# Outcomes at school-age of children born extremely preterm or extremely low birth weight in Victoria, Australia: protocol for the 8–9 years follow-up of the prospective, longitudinal Victorian Infant Collaborative Study (VICS) 2016–2017 cohort

**DOI:** 10.1136/bmjopen-2026-120087

**Published:** 2026-07-07

**Authors:** Jeanie L Y Cheong, Thi-Nhu-Ngoc Nguyen, Joy E Olsen, Lauren Pigdon, Cassidy Du Berry, Luke Howlin, Rheanna M Mainzer, Peter J Anderson, Alicia J Spittle, Lex W Doyle, The Victorian Infant Collaborative Study Group, Niranjan Abraham

**Affiliations:** 1 Prematurity Research, Clinical Sciences, Murdoch Children’s Research Institute, Parkville, Victoria, Australia; 2 Newborn Research, The Royal Women’s Hospital, Parkville, Victoria, Australia; 3 Department of Paediatrics, The University of Melbourne, Melbourne, Victoria, Australia; 4 Department of Obstetrics, Gynaecology and Newborn Health, The University of Melbourne, Melbourne, Victoria, Australia; 5 Department of Respiratory and Sleep Medicine, The Royal Children’s Hospital Melbourne, Melbourne, Victoria, Australia; 6 Respiratory Group, Murdoch Children’s Research Institute, Parkville, Victoria, Australia; 7 Clinical Epidemiology and Biostatistics Unit, Murdoch Children’s Research Institute, Parkville, Victoria, Australia; 8 Department of Pediatrics, University of California Irvine, Irvine, California, USA; 9 School of Psychological Sciences, Monash University, Melbourne, Victoria, Australia; 10 Center for Perinatal and Infant Research, Rady Children’s Health, San Diego, California, USA; 11 Department of Physiotherapy, The University of Melbourne, Melbourne, Victoria, Australia

**Keywords:** Neonatal intensive & critical care, Community child health, Infant

## Abstract

**Abstract:**

**Introduction:**

Children born extremely preterm (EP; <28 weeks’ gestation) and/or extremely low birth weight (ELBW; <1000 g) are at increased risk of difficulties across a range of neurodevelopmental domains and respiratory outcomes long term. The Victorian Infant Collaborative Study (VICS) in Australia has tracked detailed perinatal and developmental outcomes across four distinct EP/ELBW birth cohorts: 1991–1992, 1997, 2005 and 2016–2017. Improvements in 2-year developmental outcomes were reported in the VICS 2016–2017 cohort compared with previous eras; however, whether these improvements are sustained to school-age is not known. The aim of the follow-up study is to assess neurodevelopmental and respiratory outcomes at school-age in the VICS 2016–2017 cohort and examine the effect of EP/ELBW birth on school-age functioning across the VICS epochs.

**Methods and analysis:**

This is a follow-up of the VICS 2016–2017 cohort, a prospective longitudinal cohort study of children born EP/ELBW and a contemporaneous group of term-born controls born in the state of Victoria, Australia. Children were previously assessed at 2 years’ corrected age.

Follow-up at 8–9 years’ corrected age includes a comprehensive assessment of cognitive, motor, social and behavioural development and lung function.

Follow-up at 8–9 years’ corrected age includes a comprehensive assessment of cognitive, motor, social and behavioural development and lung function.

**Ethics and dissemination:**

The study has approval from the Royal Children’s Hospital Human Research Ethics Committee, Melbourne, Australia. The findings will be disseminated via peer-reviewed journal publications, conference presentations, digital and print media and through parent support/network groups.

**STRENGTHS AND LIMITATIONS OF THIS STUDY**

- One of few large longitudinal geographic cohort studies of children born extremely preterm/extremely low birth weight and contemporaneous term-born controls to examine development across childhood.
- Extensive data collection across ages from birth to 8–9 years will enable comparison of important outcomes between birth groups and between eras.
- Includes birth cohorts from the same geographical location to examine effect of neonatal intensive care on long-term development over different eras.
- Attrition at the school-age follow-up and any differences between children seen and not seen for assessment may introduce bias.
- Time and resource constraints limit the number of outcomes assessed at this follow-up.

## Introduction

 Children born extremely preterm (EP; <28 weeks’ gestation) and/or extremely low birth weight (ELBW; <1000 g) have higher rates of health and developmental difficulties than their term-born peers, including poorer lung function, more neurosensory problems, impairments in cognitive and motor development and social-emotional problems.^1^,^4^ Increased survival rates for infants born EP/ELBW are not commensurate with improvement in outcomes beyond the early childhood years^5 6^ and longitudinal research is therefore needed to understand the long-term health and development of the EP/ELBW population.

Prospective longitudinal studies of EP/ELBW children have mostly reported improvements in early childhood (age 18–36 months) outcomes for later compared with earlier birth eras. In the UK and Ireland, the EPICure studies reported increases in the rate of survival without severe disability (ie, cognitive developmental quotient (DQ) <−3 SD, non-ambulant cerebral palsy (CP), deafness or blindness) at 2.5–3 years of age from EP birth cohorts in 1995 and 2006.^7^ Similarly, the EPIPAGE studies in France reported increased survival for infants born EP without moderate-to-severe motor or sensory impairment, from 1997 to 2011.^8^ The Eunice Kennedy Shriver National Institute of Child Health and Human Development Neonatal Research Network in the USA reported improvement in neurodevelopment at 18–22 months in infants born at 22–24 weeks’ gestation over three epochs from 2000 to 2003, 2004 to 2007 and 2008 to 2011.^9^ The Victorian Infant Collaborative Study (VICS) in Australia assessed four distinct EP/ELBW birth cohorts recruited in distinct eras, that is, 1991–1992, 1997, 2005 and 2016–2017. Survival without severe developmental delay (ie, cognitive DQ <−2 SD relative to controls) or severe disability (ie, moderate-severe CP, deafness, or blindness or DQ <−2 SD) at 2 years’ corrected age had increased over the eras.^10^,^12^

However, early cognitive development is only a moderate predictor of later functioning, as skills emerge or continue to mature into middle and late childhood.^13^,^15^ The magnitude of improvements across eras in early neurodevelopmental outcomes have attenuated when the cohorts were reassessed at older ages; indeed some reports suggest that outcomes for EP/ELBW cohorts over time may be worsening. The EPICure cohorts did not observe improvements in cognitive or academic function at age 11 years between the 1995 and 2006 cohorts.^7 16^ In the VICS cohorts, the 2005 cohort displayed worse general cognitive function and academic skills at age 8 years compared with previous birth epochs of 1991–1992 and 1997.^10^ Similar trends were noted for motor and lung function, with worsening rates of non-CP motor impairment (such as developmental coordination disorder) and worsening expiratory airflow parameters over eras.^17 18^

Longitudinal studies typically enable the examination of the effect of early perinatal, biological and social factors, on later health and development of children born EP/ELBW. A serious complication of preterm birth is bronchopulmonary dysplasia (BPD) which has been associated with poor long-term lung function and neurodevelopmental outcomes.^2 19^ Since the 1990s, despite the introduction and subsequent increase in routine use of surfactant and antenatal corticosteroids, up to 50% of infants born EP develop BPD.^9 18^ With increasing use of non-invasive respiratory support to treat respiratory distress,^20^ it is important to determine whether this translates to improved expiratory airflows and developmental outcomes by school age. Social-environmental factors such as maternal education, family social demographics, parent mental health and parenting styles have been associated with cognitive and language outcomes of children born very preterm, at 5–13 years.^21^,^23^ The magnitude of association on long-term outcomes for more immature individuals, that is, those born EP/ELBW needs further clarification. Understanding key factors affecting long-term development in children born EP/ELBW will help identify those who would benefit most from support to maintain optimal developmental trajectories beyond the early years and to focus intervention on specific developmental domains which may change depending on age.

The 8–9 years follow-up of the established VICS 2016–2017 cohort will allow us to examine the effect of being born EP/ELBW on children’s neurodevelopment, respiratory health, motor and cognitive function at school age and to describe the influence of early life factors and parent mental health on development. Although this cohort had lower rates of severe developmental delay at 2 years’ corrected age compared with previous VICS cohorts from 1991 to 92, 1997 and 2005,^6 11^ it is vital to understand if improvements persist at age 8–9 years. This is particularly relevant given that we have previously reported worsening outcomes at age 8 years in the 2005 cohort compared with the earlier cohorts.^10 17 18 24^ Therefore it is imperative we assess the 2016–2017 cohort to see if the neurodevelopmental improvement at 2 years compared with earlier eras continues at an age when complex functions across motor development, cognition and academic performance can be more reliably assessed.

## Aims and hypotheses

The aim of this study is to understand the impact of EP/ELBW birth on neurodevelopment and respiratory health at 8–9 years (ie, school-age) for children born EP/ELBW in Victoria, Australia, between 2016 and 2017, and to describe how outcomes have changed compared with earlier birth cohorts.

The specific aims are:

1. To estimate the effect of being born EP/ELBW compared with being born full-term for children born EP/ELBW in Victoria, Australia, between 2016 and 2017, on outcomes at school age: cognition, behaviour, motor function, respiratory morbidity and lung function and describe the trajectories of developmental outcomes from 2 years to 8–9 years of age.
2. To describe which children born EP/ELBW are at risk of poorer school-age outcomes (ie, cognition, behaviour, motor function, respiratory morbidity and lung function), based on factors measured in pregnancy, at birth and early infancy (eg, poorer parent mental health, abnormal general movements at 3–4 months).
3. To describe the neurodevelopmental and respiratory outcomes and impairment at school-age of children born EP/ELBW across four distinct eras: 1991–1992, 1997, 2005 and 2016–17.

We hypothesise that:

1. Compared with full-term controls.

- EP/ELBW children will have poorer skills across a range of neurodevelopmental domains including general cognition, attention, memory, language and academic achievement, as well as motor function and behaviour.
- EP/ELBW children will have higher rates of wheezing illness and more airflow obstruction on lung function testing.
- EP/ELBW children will have trajectories that reflect slower development between 2 and 8–9 years, and show more variability in these trajectories compared with more stable trajectories of the full-term group.

1. Early risk factors such as fetal growth restriction, newborn respiratory problems, infection (eg, sepsis and necrotising enterocolitis), neonatal brain injury and suboptimal neurobehaviour at term-equivalent age and developmental delay at 2 years will be associated with poorer neurodevelopment and/or respiratory health at school-age. Socio-environmental risk factors (eg, lower maternal education, poorer parent mental health) will also be associated with poorer outcomes at school-age.
2. Developmental and respiratory health outcomes in the EP/ELBW group in the VICS 2016–2017 cohort will be better than those of previous birth cohorts (ie, 1991–1992, 1997 and 2005).

## Methods and analysis

### Design

Prospective longitudinal follow-up study of an established cohort.

### Study population

The VICS 2016–2017 study is a cohort study of children born EP/ELBW and a control group of children born at term and normal birth weight (37–42 weeks’ gestation at birth and ≥2500 g birth weight).

All liveborn infants born EP and/or ELBW in tertiary neonatal units in Victoria, Australia, between 1 April 2016 and 31 March 2017 (12-month period), were eligible for the study. Children were recruited during their neonatal inpatient stay. Contemporaneous controls matched with the EP/ELBW survivors according to the mother’s health insurance status (private or public, as a proxy of socioeconomic status), the main language spoken in her country of birth (English or other) and the child’s sex, were recruited shortly after birth during the same period from the maternity units affiliated with the tertiary perinatal centres. The maternity units provide general maternity care to the local population in the catchment area.

The VICS 2016–2017 recruitment criteria for EP/ELBW and term infants was identical to previous VICS cohorts, that is, 1991–1992, 1997 and 2005. Details for earlier VICS cohorts, and relevant 2-year and 8–9 years cognitive, behaviour, motor and respiratory outcomes have been published.^10 17 18 24^

#### Exclusion criteria

Similar to the previous VICS cohorts (ie, 1991–92, 1997 and 2005), live births with lethal abnormalities were excluded. Lethal anomalies were defined as major malformations, where survival was not possible even with neonatal intensive care. Term infants were excluded if they received resuscitation at birth, were admitted to neonatal intensive or special care or if they had a condition affecting neurodevelopment (eg, chromosomal anomaly).

The VICS 2016–2017 cohort was assessed at 2 years’ corrected age, and results from the perinatal, neonatal and 2-year assessments have been published.^11 20 25^ This protocol paper details the follow-up of this cohort at 8–9 years of age.

### Assessment procedure

#### Recruitment

**Figure 1:**
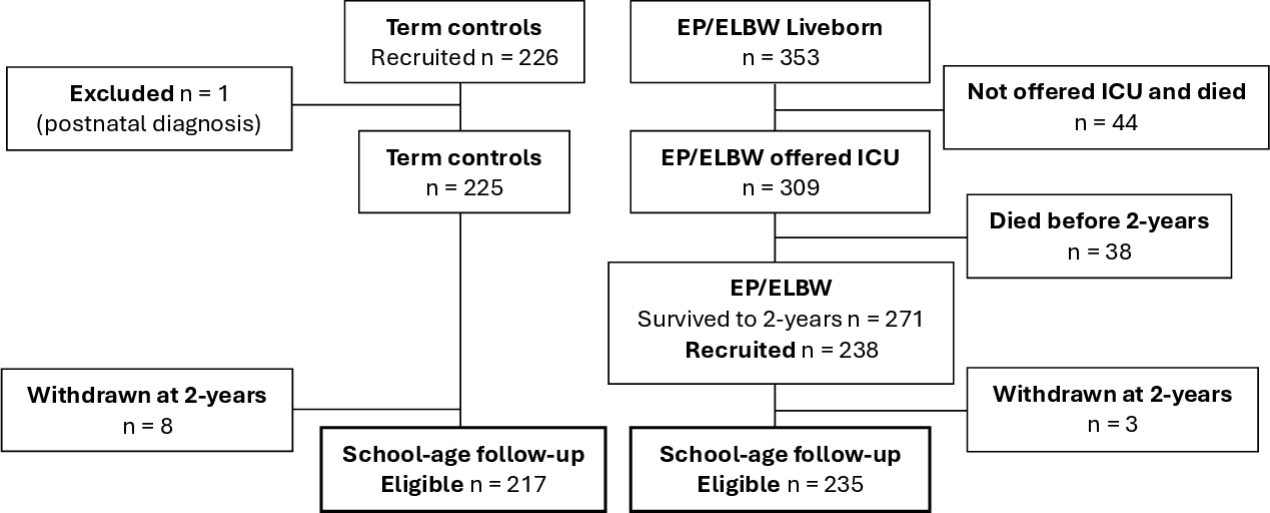
Flow chart of recruitment in VICS 2016–2017 and the eligible cohort at school-age. ELBW, extremely low birth weight; EP, extremely preterm; ICU, intensive care unit; VICS, Victorian Infant Collaborative Study.

All surviving children enrolled in the VICS 2016–2017 cohort will be eligible to participate in this follow-up study (235 EP/ELBW and 217 term-born children; participant flowchart figure 1). Parents/caregivers who consented to be approached for future research during the original study will be contacted by one of the research team and invited to participate in the school-age follow-up study. Families will be given written information about the study in the form of a plain language participant information statement booklet for parent/guardian(s) and an information sheet specifically for children. The researcher will explain all aspects of the study and families will have the opportunity to ask questions. If parent(s) agree to be in the study, written informed consent will be obtained.

#### Study visit

Participants will be invited to attend a 1-day follow-up assessment at the Murdoch Children’s Research Institute (MCRI) in Melbourne, Australia. For families who are unable to travel to MCRI, off-site in person or remote video telehealth assessments will be offered. Those who choose to have a telehealth appointment will be assessed using a modified, condensed assessment protocol that has previously been described.^26^

As part of the follow-up, participating children will have neurodevelopmental and lung function assessments. Lung function assessments will be conducted in the Pulmonary Function Laboratory at the Royal Children’s Hospital, Melbourne, by respiratory scientists. Children will have rest and meal breaks during the assessment visit. Any children who are unable to complete the assessments in 1 day will be offered another appointment on a different day.

The assessment will also include questionnaires completed online by the child’s parent/primary caregiver via a survey linked to a Research Electronic Data Capture (REDCap) database.^27^ Questionnaires will be completed prior to the assessment visit or during the assessment visit if not completed beforehand.

The assessment procedure and measures are described in more detail in the outcome measures section. Assessment measures and a timetable of the assessment sessions are outlined in tables1  2.

**Table 1:** Child neurodevelopmental assessment measures

Assessment	Domain	Description
WISC-V^28^	General cognitive function (IQ)	The WISC-V is considered a gold standard measure for assessing general intelligence in children aged 6–16 years. The 10 core subtests are administered to provide scores for 5 indices and an estimate of full-scale IQ.
CVLT-C^42^	Verbal learning and retention	The CVLT-C is standardised for participants aged 5–16 years and assesses verbal learning, free recall and memory.
TEA-Ch2^43^	Sustained attention	Sustained attention will be assessed with the Score! subtest of the TEA-Ch. The TEA-Ch is a sensitive measure of attention with norms for ages 6–16 years.
Contingency Naming Test^44^	Executive function: mental flexibility	Assesses aspects of executive function (response inhibition, working memory and mental flexibility). Consists of four trials of increasing task difficulty where the participant names shapes and colours according to learnt rules.
RCF^45^	Executive function: planning and organisation	Aspects of executive function (visuospatial organisation and planning) will be assessed using the copy trial of the RCF which involves reproducing a complicated line drawing that is scored for accuracy and strategy.
WRAT5^46^	Academic achievement	The WRAT5 is an age-standardised test of academic achievement in participants aged 5 years and above. Three selected subtests from the WRAT5 will assess reading, spelling and math computation.
MABC-3^29^	Fine and gross motor function	The MABC-3 is considered the gold standard assessment of motor ability and impairment from 3 to 25 years of age. It is divided into three age-standardised subscales: Manual Dexterity, Aiming and Catching and Balance and Locomotion.

CVLT-C, California Verbal Learning Test – Children’s Version; MABC-3, Movement Assessment Battery for Children, third edition; RCF, Rey Complex Figure Test; TEA-Ch2, Test of Everyday Attention for Children, second edition; WISC-V, Wechsler Intelligence Scale for Children, fifth edition; WRAT5, Wide Range Achievement Test, fifth edition.

**Table 2:** Assessment timetable and breaks

Session description	Time (minutes)
Session 1 cognitive assessments	75–90
Morning tea break	15
Session 2 lung function and anthropometry	90
Lunch break	30
Session 3 cognitive assessments	60
Afternoon tea break	15
Session 4 motor assessment	30

Data collection commenced in October 2024 and is planned to finish in October 2026.

### Outcome measures

All assessments will be conducted by trained professionals who are unaware of participant group allocation (birth status), clinical history and previous assessment results. Assessment measures are outlined in table 1.

### Neurodevelopmental assessments

#### Cognition

General cognitive ability will be assessed using the Wechsler Intelligence Scale for Children-fifth Edition^28^ for an estimate of full-scale IQ and five domain scores: verbal comprehension, visual spatial, fluid reasoning, working memory and processing speed. Other assessments will be administered to assess various cognitive domains including attention, learning and memory, executive function and academic achievement (table 1).

### Motor and physical function

#### Fine and gross motor skills

Fine and gross motor skills will be assessed using the Movement Assessment Battery for Children - third edition.^29^ The assessment is divided into three age-standardised subscales of Manual Dexterity, Aiming and Catching and Balance and Locomotion and yields a Total Motor score which can be used to classify motor impairment based on score percentiles.

#### Cerebral palsy

For children with a diagnosis of CP the Gross Motor Function Classification System (GMFCS)^30^ will be used to classify motor function. The GMFCS is a reliable and widely used scale that classifies children’s gross motor function based on their self-initiated movement. Parents will also be asked for permission to access their child’s diagnosis information from the Australian CP Register, a national database that includes clinical information on each person with CP from relevant electronic medical records.

#### Anthropometry and blood pressure

Growth measurements, including height and weight, will be recorded without shoes to the nearest 0.1 cm and 0.1 kg on an ultrasonic stadiometer with digital scales (Seca 285). Additional circumferential and segmental anthropometric measures will be obtained using a tape measure to the nearest 0.1 cm, in line with WHO standards.^31 32^ Systolic and diastolic blood pressure estimates, and heart rate and mean arterial pressure, will be obtained non-invasively using an automated oscillometric device (Connex VSM 6000), with measurement performed in accordance with American Academy of Pediatrics clinical practice guidelines.^33^ Measurements will be taken from the right arm after ≥5 min seated rest, using an appropriately sized cuff determined by mid-arm circumference measured prior.

Anthropometric outcomes will be converted to SD scores using the UK-WHO preterm growth references,^34^ while blood pressure outcomes will be analysed relative to the local term-born control cohort.

### Parent questionnaires

Parents will be asked to complete questionnaires about their child’s health and development, social demographics and their own mental health (table 3).

**Table 3:** Parent questionnaires

Questionnaire	Domain	Description
BRIEF-2^47^	Executive function behaviours	The BRIEF-2 consists of 63 items scored across 9 clinical factors, and yields 3 composite index scores, estimating behaviour, emotion and cognition, and an overall global executive function score for children aged 5–18 years.
SDQ^48^	Emotional and behavioural skills	The SDQ is a widely used screening tool that consists of 25 items assessing emotion and behavioural problems for children aged 4–16 years.
SCQ^49^	Autism symptoms	The SCQ is a 40-item screening tool that is well-validated and used to assess communication skills and social functioning specific to autism spectrum disorders for general and clinical populations aged 4 years and older.
ADHD-RS^50^	Attention	The ADHD-RS consists of 18 items and is a valid and reliable instrument widely used by clinicians in screening, diagnosis and treatment evaluation of ADHD.
DCDQ’07^51^	Movement and coordination	The DCDQ’07 is a 15-item parent-reported measure designed to identify subtle motor problems in children and provides a score for 3 subscales (control during movement, fine motor and general coordination) as well as a total score.
PedsQL^52^	Quality of life	The PedsQL is a 23-item parent questionnaire designed to assess parents’ perceptions of their child’s health and quality of life. The Core Scales for children aged 8–12 years encompasses four subscales: physical, emotional, social and school functioning, as well as a total scale score.
ISAAC core questionnaire^53^	Asthma and allergies	The ISAAC core questionnaire is a 15-item parent-reported questionnaire that is widely used by respiratory practitioners to assess symptoms of asthma, allergic rhinitis and eczema.
GAD-7^35^	Parent mental health: anxiety symptoms	The GAD-7 is a valid and reliable 7-item screening tool for anxiety symptoms and assessing its severity in clinical and research settings.
CESD-R^36^	Parent mental health: depression symptoms	The CESD-R is a well validated and reliable 20-item questionnaire that measures symptoms of depression in general and clinical populations.
Social background questionnaire	Social background and demographics	A demographic questionnaire (study-developed items) will include questions about family structure, employment and education and language spoken at home.

ADHD-RS, Attention Deficit/Hyperactivity Disorder Rating Scale, fifth edition; BRIEF-2, Behaviour Rating Inventory of Executive Function, second edition; CESD-R, Centre for Epidemiologic Studies Depression Scale – Revised; DCDQ’07, Developmental Coordination Disorder Questionnaire; GAD-7, Generalised Anxiety Disorder 7-item; ISAAC, International Study of Asthma and Allergies in Childhood; PedsQL, Paediatric Quality of Life Inventory Quality of Life Inventory; SCQ, Social Communication Questionnaire; SDQ, Strengths and Difficulties Questionnaire.

#### Child health and development

The questionnaires include four measures of child behaviour and communication, and parent report of their child’s motor coordination, health and quality of life and respiratory symptoms.

Family demographic data (such as maternal education and primary languages spoken at home) will be used to determine socioeconomic status as per previous VICS cohorts.

#### Parent/caregiver mental health

Parents will be asked to complete the Generalised Anxiety Disorder 7-item^35^ and Centre for Epidemiologic Studies Depression Scale – Revised.^36^ Both questionnaires will be scored contemporaneously with the child assessments via REDCap, and parents scoring in the at-risk range for either assessment will be contacted by a psychologist from the research team to explore their current support needs, and provide information on available services if required.

### Lung function

Lung function will be assessed at an accredited pulmonary function laboratory by a trained paediatric respiratory scientist blinded to the child’s birth group. Lung function tests and variables are listed in table 4. Spirometry will be conducted pre-bronchodilator and post-bronchodilator in accordance with European Respiratory Society (ERS) and American Thoracic Society (ATS) guidelines.^37^ Relevant Global Lung Initiative predictive equations will be used to generate z-score measures.^38^ Static lung volumes will be assessed via whole-body plethysmography, while rapid gas analysers connected to a spirometer will be used to determine the diffusion capacity of the lungs for carbon monoxide (DL_CO_). Nitrogen multiple-breath washout will be used to determine the Lung Clearance Index, a global marker of ventilation inhomogeneity that is more sensitive than traditional measures of expiratory airflow for the detection of early small airway dysfunction. ^39^Airwave oscillometry, a test that measures the impediment to moving air in and out of the lungs via the application of oscillatory pressure signals at the mouth during regular quiet breathing, will be used to evaluate resistance and reactance and infer mechanical properties of the pulmonary system, such as stiffness and elastance.

**Table 4:** Lung function measures and variables

Lung function	Variable measured
Airflow	FEV_1_: forced expired volume in 1 s FVC: forced vital capacity FEV_1_/FVC FEF_25-75%_: forced expiratory flow at 25–75% of the FVC
Lung volumes	TLC: total lung capacity FRC: functional residual capacity ERV: expiratory reserve volume RV: residual volume RV/TLC
Reversibility	Bronchodilator to determine how much airflow obstruction is reversible: Post bronchodilator FEV_1_ Post bronchodilator FVC Post bronchodilator FEV_1_/FVC Post bronchodilator FEF_25-75%_
Gas exchange	DL_co_: diffusing capacity of the lung for carbon monoxide, which reflects gas transfer across the alveolar-capillary interface Va: alveolar volume K_CO_: transfer coefficient of carbon monoxide
Ventilation inhomogeneity	LCl_2.5_: lung Clearance Index at 2.5% S_cond_ (airway conductance) S_acin_ (acinar conductance)
Oscillometry	Respiratory resistance at 5 Hz (R_rs5_) Resonant frequency (Fres) Area under the reactance curve (AX) Respiratory system reactance at 5 Hz (X_rs5_) Index of frequency dependence of respiratory system resistance between 5 Hz and 20 Hz (R_rs5-20_)

### Assessment feedback for participants

Families will be given a written report with the results from the cognitive, motor and lung function assessments. Results will be presented in terms of performance ranges (ie, below average, average, above average) for cognitive and motor assessments. ERS/ATS guidelines will be used to quantify and report the presence and severity of lung function abnormalities.^37^

If there are any assessment findings that require clinical follow-up, including parents’ responses on the mental health questionnaires, the families will be contacted by the research team to discuss the assessment results and referral options to appropriate services as indicated.

### Sample size

The study sample size is determined by the number of eligible participants enrolled in the VICS 2016–17 cohort (EP/ELBW, n=235; full-term, n=217). Based on follow-up rates at the 2-year assessment (92% EP/ELBW; 90% controls), we conservatively estimate an 85% retention rate at school-age for the eligible VICS 2016–2017 cohort. This will result in an estimated sample of 199 children born EP/ELBW and 184 term-born control children at 8–9 years (total n=383). Assuming 15% of participants are twins and an intraclass correlation coefficient of 0.6 between outcomes for twins, this will result in effective sample sizes of 187 EP/ELBW and 173 controls, enabling us to detect difference in outcomes between the groups as small as 0.3 SDs with 80% power (based on a two-sided test with α=0.05). For an estimated effect of 0.3 SDs, with sample sizes of 187 EP/ELBW and 173 controls, a 95% CI will be from 0.09 to 0.51. An effect size of 0.3 SDs is reasonable because this is a clinically important difference. For proportions, if the event rate is 10% in the controls (eg, for anxiety or depression), we will have 76% power to detect an absolute increase of 10% in the EP/ELBW group (event rate of 20%).

### Data management

All outcome data from the neuropsychology, motor and lung function assessments will be entered into an MCRI REDCap database by research team members. Parent questionnaire responses will be linked to the REDCap database via online surveys. Electronic data will be securely stored in MCRI’s REDCap database system and in files stored on MCRI’s network file servers, which are backed up daily. Data will be securely stored until the youngest participant reaches the age of 25 years or for 15 years after the study has been complete, whichever is later, unless further follow-up of the cohort is planned.

### Statistical analysis

Data will be analysed according to the aims of the study.

#### Aim 1

The effect of being born EP/ELBW compared with being born at term on outcomes (eg, cognition, lung function) at age 8–9 years will be estimated using generalised linear models, with adjustment for confounders (eg, social demographics, medical history). Estimands will be specified using the ‘target trial’ approach.^40^ Directed acyclic graphs will be developed using expert knowledge and current literature to depict our assumptions about causal relationships between variables and inform confounder selection. If warranted, g-methods will be used for estimation. Models will be fitted using generalised estimating equations (GEEs) to account for clustering by multiples (eg, twins/triplets).

Mean trajectories of developmental outcomes (eg, cognition) will be compared between EP/ELBW and full-term controls using mixed effects models. Models will include fixed effects of time, group and a time-by-group interaction and a random intercept (and a random slope if warranted), to allow for repeated measures within individuals.

#### Aim 2

Mean outcomes (eg, neurodevelopment, respiratory health) at age 8–9 years will be contrasted between subgroups of children born EP/ELBW defined by factors measured in pregnancy and early infancy. Contrasts (eg, difference in means) will be estimated using univariable generalised linear models, fitted using GEEs to account for clustering of multiples.

#### Aim 3

Difference in mean outcomes at age 8–9 years across VICS eras (1991–1992, 1997, 2005 and 2016–2017) will be estimated using univariable generalised linear models, fitted using GEEs to account for clustering of multiples, with the VICS 2016–2017 cohort as the reference. Comparisons will be presented as risk differences or mean differences.

#### Missing data

For all aims, we will take a principled approach to handling missing data. Directed acyclic graphs will be used to outline our assumptions regarding the causes of missingness, which will be used to guide the analytical approach (eg, multiple imputation or delta-adjusted sensitivity analyses where warranted).

### Patient and public involvement

The project aligns with the priority research areas identified in our Delphi study of parents with experience of newborn medicine, which includes addressing long-term impacts on child health and development.^41^ The study protocol and participant documentation had input from those with lived experience of preterm birth. We intend to seek input from members of our Lived Experience Network from the Australia National Health and Medical Research Council Extremely Preterm Infant Centre of Research Excellence, with dissemination of research findings at different forums to ensure maximum reach.

### Ethics and dissemination

The study has ethics approval from The Royal Children’s Hospital Human Research Ethics Committee, Melbourne, Australia (HREC 35237/15/RCHM/110) with governance approval from all participating sites. Parents will provide written informed consent for their child and themselves to participate. Study findings will be disseminated through publications in peer-reviewed journals, conference presentations and via digital and print media. Results will be disseminated to participants and key stakeholders, facilitated by the Lived Experience Network in our Extremely Preterm Infant Centre of Research Excellence, on the VICS study webpage and through parent support groups.
