# Supplementary material for: Outcomes at school-age of children born extremely preterm or extremely low birth weight in Victoria, Australia: protocol for the 8–9 years follow-up of the prospective, longitudinal Victorian Infant Collaborative Study (VICS) 2016–2017 cohort
Source: BMJ Open. 2026 Jul 7;16(7):e120087. doi: 10.1136/bmjopen-2026-120087 (PMC13343067; doi:10.1136/bmjopen-2026-120087)
Supplement: Supplementary file 1 [file bmjopen-2026-120087.reviewer_comments.pdf]

PEER REVIEW HISTORY

BMJ Open publishes all reviews undertaken for accepted manuscripts. Reviewers are asked to complete a checklist review form and are provided with free text boxes to elaborate on their assessment. These free text comments are reproduced below.

ARTICLE DETAILS

Title (Provisional)

Outcomes at school-age of children born extremely preterm or extremely low birth weight in Victoria, Australia: protocol for the 8-9 year follow-up of the prospective, longitudinal Victorian Infant Collaborative Study (VICS) 2016-17 cohort

Authors

Cheong, Jeanie; Nguyen, Thi-Nhu-Ngoc; Olsen , Joy E; Pigdon, Lauren; Du Berry, Cassidy; Howlin, Luke; Mainzer, Rheanna; Anderson, Peter J; Spittle, Alicia J; Doyle, Lex William

---

VERSION 1 - REVIEW

**Reviewer**	**1**
**Name**	**Spiegler, Juliane**
**Affiliation**	**Universitatsklinikum Wurzburg**
**Date**	**12-Apr-2026**
**COI**	**None**

Thank you for preparation of this study protocol.

I find it well written and I have nothing to add. I am looking forward to the results from this follow-up!

**Reviewer**	**2**
**Name**	**Finken, Martijn**
**Affiliation**	**Amsterdam UMC**
**Date**	**19-May-2026**
**COI**	**None**

This manuscript is a description of a study protocol for of a new wave of investigations at age 9 years of the Victorian Infant Collaborative Study 2016-17 cohort.

I have few comments:

1. Study population (p7): Please, comment on the representativeness of the control population. The manuscript states that controls "were recruited shortly after birth during the same period from the maternity units affiliated with the tertiary perinatal centres". In fact, there is mostly a reason for giving birth in a specialized center. In which respects deviated controls from the general population? In other words, were controls relatively healthy or relatively affected?

2. Recruitment (p8): Please, specify the recruitment procedure. Research team members can only contact parents who have previously granted permission to be approached for future research.

3. Study visit (p8): Please, include a time table for the study procedures, including brakes. The study is time-consuming for 9-year-olds. Has the feasibility of the study protocol been tested at this age?

4. Anthropometry and blood pressure (p10): Please, rewrite this part. I guess that height, weight and head circumference, unlike fat (free) mass, are not measured by a body composition analyzer. In which position and after how much rest was blood pressure measured? Did you adhere to international standard for blood pressure measurement? How will you deal with these data? I guess that you convert both anthropometric and blood pressure data to standard deviation score, but which population references will you use?

VERSION 1 - AUTHOR RESPONSE

Reviewers' comments:
Reviewer #1:
Thank you for preparation of this study protocol.
I find it well written and I have nothing to add. I am looking forward to the results from this follow-up!

Thank you for reviewing the protocol and for your comments.

Reviewer #2: This manuscript is a description of a study protocol for of a new wave of investigations at age 9 years of the Victorian Infant Collaborative Study 2016-17 cohort.

I have few comments:
1. Study population (p7): Please, comment on the representativeness of the control population. The manuscript states that controls "were recruited shortly after birth during the same period from the maternity units affiliated with the tertiary perinatal centres". In fact, there is mostly a reason for giving birth in a specialized center. In which respects deviated controls from the general population? In other words, were controls relatively healthy or relatively affected?

Term controls are representative of the local population within the hospital catchment areas. The hospital maternity units affiliated with the tertiary perinatal centres provide general maternity care for the population in the geographic region of the tertiary perinatal centres. Term controls were normal birth weight (37-42 weeks' gestation at birth and ≥2500g birthweight), and healthy, and were excluded if they received resuscitation at birth, were admitted to neonatal intensive or special

care, or if they had a condition affecting neurodevelopment (e.g., chromosomal anomaly) (page 7, Study population section, paragraphs 1 and 3).

We have added the statement in brackets (page 7, Study population section, paragraph 1):

"…were recruited shortly after birth during the same period from the maternity units affiliated with the tertiary perinatal centres. (The maternity units provide general maternity care to the local population in the catchment area.)"

2. Recruitment (p8): Please, specify the recruitment procedure. Research team members can only contact parents who have previously granted permission to be approached for future research.

Parents were contacted if they had agreed to be approached for future research when consenting to the original study.

We have added to the sentence in the main text (page 8, Assessment procedure section, paragraph 1):

"Parents/caregivers who consented to be approached for future research during the original study will be contacted by one of the research team and invited to participate in the school-age follow-up study"

3. Study visit (p8): Please, include a time table for the study procedures, including brakes. The study is time-consuming for 9-year-olds. Has the feasibility of the study protocol been tested at this age?

We have adapted the protocol based on our experience of other follow-up studies of school-aged children.[1 2 3] We have also conducted mock sessions with children of similar age (volunteers known to the research team) to test the feasibility and acceptability for children and parents, and have discussed the protocol with members of our Lived Experience Network, many of whom are parents of children born preterm. For any children who are unable to complete the assessment in one day, they will be offered another assessment session on a different day.

We have revised the section of the manuscript to state (page 8-9, Study visit section, paragraph 2):

"As part of the follow-up, participating children will have neurodevelopmental and lung function assessments. Lung function assessments will be conducted in the Pulmonary Function Laboratory at the Royal Children's Hospital, Melbourne, by respiratory scientists. Children will have rest and meal breaks during the assessment visit. Any children who are unable to complete the assessments in one day will be offered another appointment on a different day."

We have added the following statement (page 9, Study visit section, paragraph 4) and timetable of assessments (Table 2, page 12):

"Assessment measures and a timetable of the assessment sessions are outlined in Table 1 and Table 2."

Table 2. Assessment timetable and breaks

Session description		**Time** (minutes)
Session 1	Cognitive assessments	75-90
	Morning tea break	15

Session 2 Lung function and anthropometry	90
Lunch break	30
Session 3 Cognitive assessments	60
Afternoon tea break	15
Session 4 Motor assessment	30

Tables 2 and 3 have been renamed Table 3 and 4 (pages 13 and 14).

4. Anthropometry and blood pressure (p10): Please, rewrite this part. I guess that height, weight and head circumference, unlike fat (free) mass, are not measured by a body composition analyzer. In which position and after how much rest was blood pressure measured? Did you adhere to international standard for blood pressure measurement? How will you deal with these data? I guess that you convert both anthropometric and blood pressure data to standard deviation score, but which population references will you use?

We will use the UK-WHO preterm growth references[4] for anthropometric data, and the local term control data for blood pressure measurements. Anthropometric data measurements will be collected according to WHO standards.[5 6] Blood pressure measures will be collected via non-invasive oscillometric methods, as per American Academy of Pediatrics clinical practice guidelines.[7]

We have revised the section on anthropometry and blood pressure (page 10-11, paragraph 1 and 2):

"Growth measurements, including height and weight, will be recorded without shoes to the nearest 0.1cm and 0.1kg on an ultrasonic stadiometer with digital scales (Seca 285). Additional circumferential and segmental anthropometric measures will be obtained using a tape measure to the nearest 0.1cm, in line with WHO standards.[5 6] Systolic and diastolic blood pressure estimates, and heart rate and mean arterial pressure, will be obtained non-invasively using an automated oscillometric device (Connex VSM 6000), with measurement performed in accordance with American Academy of Pediatrics clinical practice guidelines.[7] Measurements will be taken from the right arm after ≥5 minutes seated rest, using an appropriately sized cuff determined by mid-arm circumference measured prior.

Anthropometric outcomes will be converted to standard deviation scores using the UK-WHO preterm growth references,[4] while blood pressure outcomes will be analysed relative to the local term-born control cohort."

References

1. Cheong JLY, Anderson PJ, Burnett AC, et al. Changing Neurodevelopment at 8 Years in Children Born Extremely Preterm Since the 1990s. *Pediatrics* 2017;139(6) doi: 10.1542/peds.2016-4086
2. Burnett AC, Anderson PJ, Lee KJ, et al. Trends in executive functioning in extremely preterm children across 3 birth eras. *Pediatrics* 2018;141(1):e20171958. doi: 10.1542/peds.2017-1958
3. Spittle AJ, Cameron K, Doyle LW, et al. Motor impairment trends in extremely preterm children: 1991–2005. *Pediatrics* 2018;141(4):e20173410. doi: doi.org/10.1542/peds.2017-3410
4. Cole TJ, Williams AF, Wright CM, et al. Revised birth centiles for weight, length and head circumference in the UK-WHO growth charts. *Ann Hum Biol* 2011;38(1):7-11. doi: 10.3109/03014460.2011.544139

5. World Health Organization. Training Course on Child Growth Assessment. WHO Child Growth Standards Training Course. Geneva, 2008.
6. World Health Organization. Expert Committee on Physical Status. Physical Status: The use and interpretation of anthorpometry. Report of a WHO Expert Committee. WHO Technical Report Series. Geneva, 1995:463.
7. Flynn JT, Kaelber DC, Baker-Smith CM, et al. Clinical Practice Guideline for Screening and Management of High Blood Pressure in Children and Adolescents. *Pediatrics* 2017;140(3) doi: 10.1542/peds.2017-1904

VERSION 2 - REVIEW

**Reviewer**	**2**
**Name**	**Finken, Martijn**
**Affiliation**	**Amsterdam UMC**
**Date**	**16-Jun-2026**
**COI**	

All my comments were satisfactorily addressd by the authors!
